# The Association Between Adverse Childhood Experiences and Sleep in Children with Autism Spectrum Disorder

**DOI:** 10.1007/s10803-024-06321-6

**Published:** 2024-07-06

**Authors:** Eleonora Sadikova, Micah O. Mazurek

**Affiliations:** University of Virginia, 417 Emmet Street South, Charlottesville, VA 22904 USA

**Keywords:** Autism spectrum disorder, Adverse childhood experiences, Trauma, Sleep, National survey of children’s health

## Abstract

Children with autism spectrum disorder are at higher risk for adverse childhood experiences (ACEs). They are also more vulnerable to sleep problems and are less likely to obtain the recommended number of hours of sleep than neurotypical children. In the general population, ACEs have been linked to future sleep difficulties. Despite increased vulnerabilities to both ACEs and sleep problems, no study has examined this association in ASD. Using the National Survey of Children’s Health across four cohorts, we examined whether ACEs were a risk factor to obtaining the recommended number of hours of sleep, while accounting for demographic and health factors typically associated with sleep duration. Findings indicate that children with ASD with more ACEs were less likely to get the recommended number of hours of sleep than children with fewer ACEs. Other factors associated with sleep included race, anxiety, autism severity, and overall health. These findings indicate that sleep problems in children with ASD are complex and multifaceted. Among other considerations, it is important for clinicians to screen children with ASD for ACEs and consider the possible impact of ACEs on sleep.

Autism spectrum disorder (ASD) occurs in about 1 in 44 children (or 2.27%; Maenner, 2021), with increasing global prevalence every year (Zeidan et al., 2022). ASD is classified by deficits in social interaction and social communication and by the presence of restricted and repetitive behaviors and interests (American Psychiatric Association, 2013). In addition to these diagnostic features, ASD is also associated with many co-occurring medical and mental health conditions (Soke et al., 2018). Sleep difficulties are among the most prevalent of these co-occurring challenges for children with ASD.^1^ Up to 80% of children with ASD experience sleep difficulties (Aathira et al., 2017; Reynolds & Malow, 2011; Richdale & Schreck, 2009). Sleep problems in children with ASD vary greatly in nature, and include difficulties falling sleep (e.g., bedtime resistance), night wakings, parasomnias (e.g., sleep walking), night terrors, and sleep disordered breathing (e.g., sleep apneaRichdale & Schreck, 2009; Schreck & Richdale, 2020). Notably, obstructive sleep apnea (OSA) is common in children with ASD (Tomkies et al., 2019), who experience more OSA symptoms than neurotypical children (Santapuram et al., 2022). The direct outcome of most of these sleep problems is an overall decrease of sleep duration, or insomnia.

Sleep-related problems (e.g., insufficient sleep, sleep disordered breathing) are associated with numerous developmental challenges in children with ASD including increased severity of ASD symptoms (Hundley et al., 2016; Sadikova et al., 2022), anxiety (Mazurek & Petroski, 2015), hyperactivity, inattention, irritability, and aggression (Mazurek & Sohl, 2016). In a recent metanalysis conducted by Han and colleagues (2022), of 15,074 autistic individuals from 49 different studies, sleep difficulties in autistic individuals were associated with worse daytime functioning and clinical symptomatology, including internalizing and externalizing behaviors, executive functioning difficulties, and ASD symptom severity.

The mechanisms that contribute to the high rates of co-occurrence of sleep difficulties in children with ASD are not well known. Sleep disordered breathing may be a part of this mechanism, as children with ASD are more vulnerable to experiencing sleep disordered breathing (Tomkies et al., 2019). In fact, treating obstructive sleep apnea in children with ASD using adenotonsillectomy may result in improvement in behavioral problems (Murata et al., 2017). Other mechanisms may be related to sensory responsivity and anxiety (Mazurek & Petroski, 2015). Mazurek and Petroski (2015) posited that children with sensory sensitivities and anxiety may have increased physiological arousal, which may lead to more difficulty falling and staying asleep (Mazurek & Petroski, 2015). Hyperarousal plays an important role in the development and maintenance of insomnia in the general population (Vargas et al., 2020), Insomnia frequently includes sleep disordered breathing (Krakow, 2022; Krakow et al., 2019), and many with sleep disordered breathing have co-occurring anxiety (Garbarino et al., 2020). There is increasing evidence for the link between hyperarousal and sleep problems in children with ASD (Harder et al., 2016; Taylor & Corbett, 2014).

Notably, adverse and traumatic experiences also cause hyperarousal and sleep disruption in the general population (Kolk, 2003). In fact, adverse childhood experiences are well-established predictors of sleep difficulties in the general population (Charles et al., 2022; Vadukapuram et al., 2022). Adverse childhood experiences (ACEs) are stressful or traumatic experiences that occur in childhood, such as maltreatment or household dysfunction (Cronholm et al., 2015; Felitti et al., 1998). ACEs have long-term developmental effects on physical and mental health (Anda et al., 2006; Monnat & Chandler, 2015), and predict later development of sleep problems (Kajeepeta et al., 2015). Trauma is also bi-directionally associated with obstructive sleep apnea (Colvonen et al., 2019). Notably, children with ASD are more vulnerable to experiencing ACEs (Hoover & Kaufman, 2018; Rigles, 2021). Despite children with autism being more vulnerable to experiencing ACEs and having more sleep difficulties than neurotypical children, the link between ACEs and sleep has not been previously investigated in this population.

The aim of this study was to examine the association between ACEs and sleep duration in a large national sample of children with ASD, while accounting for other established associations with sleep duration in this population such as age, sex, household income, ASD severity, anxiety, depression, and overall physical health.

## Materials and Methods

Analyses were conducted using data from the National Survey of Children’s Health (NSCH; United States Census Bureau, 2016, 2017, 2018, 2019). The NSCH is a nationally distributed survey of children across the United States. Randomly sampled households were invited to complete an online survey if they had children under 18 residing in the home. Children with special healthcare needs were intentionally oversampled. Although the survey is distributed each year, data are cross-sectional, meaning that information is not collected on the same individuals over time. Data from 4 waves of the NSCH were included in the current study. Out of 131,774 caregiver respondents, 3,247 (2.46%) reported that their child was diagnosed with autism by a healthcare professional, and were thereby included in the analyses.

### Measures

All variables used were based on parent/caregiver report on the NSCH.

#### Demographic Information

Age, sex, race, and household income was collected and accounted for in the analyses. Household income was measured as the percentage of the Federal Poverty Level (FPL).

#### Autism Diagnosis and Severity

Parents/caregivers were asked whether a healthcare professional had ever diagnosed their child with autism spectrum disorder, Asperger’s, autistic disorder, or pervasive developmental disorder. Children who had ever been diagnosed with any of those were included in the sample. Parents who said “yes” were also asked to rate their child’s autism severity as mild, moderate, or severe.

#### Anxiety

Parents/caregivers reported whether their child had current anxiety problems diagnosed by a healthcare professional.

#### Depression

Parents/caregivers were also asked whether their child had current depression diagnosed by a healthcare professional.

#### Behavior or Conduct Problems

Parents/caregivers were asked whether their child had current behavior or conduct problems identified by an educator or healthcare provider.

#### Overall Health

Parents/caregivers were asked to rate their child’s overall health as Excellent, Very Good, Good, Fair, or Poor.

#### Sleep

Parents/caregivers were asked how many hours of sleep their child received during an average day over the past week (including both nighttime sleep and naps): Less than 6 h, 6 h, 7 h, 8 h, 9, hours, 10 h, 11 or more hours. These selections were then converted to a binary variable in the on whether the child gets an adequate number of hours of sleep per the American Academy of Pediatrics recommendations in the NSCH Codebook. This approach was selected because the developmental expectations for adequate sleep change substantially throughout development.

#### Adverse Childhood Experiences

Parents and caregivers responded to a series of questions about whether or not their child had experienced specific incidents: (1) parent or guardian divorced or separated, (2) parent or guardian died, (3) parent or guardian served time in jail, (4) saw or heard parents or adults slap, hit, kick, punch one another in the home, (5) was a victim of violence or witnessed violence in his or her neighborhood, (6) lived with anyone who was mentally ill, suicidal, or severely depressed, (7) lived with anyone who had a problem with alcohol or drugs, or (8) treated or judged unfairly because of his or her race or ethnic group. The adverse childhood experiences were then summed for analyses.

### Statistical Analyses

Analyses were conducted with Stata/IC 16.1. We used a hierarchical binary logistic regression to examine the association between sleep and ACEs. Covariates and variables of interest were entered in three blocks. In the first block, we entered demographic variables including age, sex, household income, and race. In the second block, we entered mental and physical health factors associated with sleep as indicated by previous literature. The second block included autism severity, anxiety, depression, behavior and conduct problems, and overall health. In the third and final block, we entered the number of ACEs experienced (range 0–8). For each block, Wald tests were used to determine whether there was improved model fit with each addition.

## Results

For sample characteristics, see Table 1. The overall model showed a better fit than the null model (χ^2^ (15) = 95.41, p < 0.001). Wald tests indicated that demographic variables were significantly associated with getting the recommended number of hours of sleep (Wald (9) = 60.54, p < 0.001). The second block of mental and physical health indications also significantly improved model fit (Wald (5) = 27.06, p < 0.001). Finally, the last block showed that adding ACEs improved model fit even further above and beyond already included variables (Wald (1) = 4.87, p = 0.027).

**Table 1:** Sample characteristics and descriptive statistics (*N* = 3,247)

	Frequency (%)
Sex (male)	2,602 (80.4)
Race	
White	2,495 (76.8)
Black	249 (7.7)
American indian or alaska native	34 (1.1)
Asian	131 (4.3)
Native hawaiian or other pacific islander	9 (0.3)
Other	60 (1.9)
Two or more races	269 (8.3)
Federal poverty level (FPL) percentage	
0–99% FPL	524 (16.14)
100–199% FPL	655 (20.17)
200–399% FPL	987 (30.40)
400 FPL or above	1,081 (33.29)
Overall health	
Excellent	1,089 (33.6)
Very good	1,203 (37.1)
Good	752 (22.3)
Fair	195 (6.0)
Poor	29 (0.9)
Autism severity	
Mild	1,643 (51.09)
Moderate or severe	1,573 (48.91)
Mental health	
Anxiety	1,504 (47.2)
Depression	519 (16.3)
Behavior problems	1,747 (55.6)
Adverse childhood experiences	
None	1,350 (41.8)
One	845 (26.1)
Two	491 (15.2)
Three	222 (6.9)
Four	154 (4.8)
Five	92 (2.8)
Six	47 (1.5)
Seven	24 (0.7)
Eight	8 (0.2)
	M (SD)
Age (range: 3–17 years)	11.32 (4.08)

In the final model with all blocks, age was significantly associated with getting the recommended number of hours of sleep, and older children were 6% more likely to obtain the recommended number of hours of sleep (*OR* = 1.06, 95% CI: 1.03 – 1.09). Sex was not associated with getting the recommended number of hours of sleep. Race was associated with getting recommended number of sleep, as Black children were 48% less likely to receive the recommended number of hours of sleep than White children (*OR* = 0.52, 95% CI: 0.34–0.78). Federal poverty level was statistically but not meaningfully associated with sleep duration (*OR* = 1.00, 95% CI: 1.000 – 1.002). Parent-rated overall health of the child was associated with getting the recommended sleep duration as those with poorer health were 16% less likely to get the recommended number of hours of sleep than those with better health (*OR* = 0.84, 95% CI: 0.74–0.95). Similarly, children with moderate or severe ASD were 26% less likely to get the recommended number of hours of sleep than those with mild parent-reported ASD (*OR* = 0.74, 95% CI: 0.60–0.93). Anxiety, behavior problems, and depression were not associated with sleep problems. Finally, after accounting for all other variables, children with more ACEs were 9% less likely to get the recommended number of hours of sleep than children with fewer ACEs (*OR* = 0.91, 95% CI: 0.84–0.96). Odds ratios and significance level for all the models and variables are presented in Table 2.

**Table 2:** Summary of hierarchical binary logistic regression analysis for variables predicting getting recommended number of hours of sleep

	Model 1 odds ratio	Model 2 odds ratio	Model 3 odds ratio
Age	1.05***	1.05***	1.06***
Sex: female	1.20	1.21	1.23
Race			
Black or african american	0.51**	0.51**	0.52**
American indian or alaska native	0.67	0.73	0.77
Asian	0.59	0.61	0.59
Native hawaiian or pacific islander	0.39	0.36	0.35
Other	0.74	0.75	0.75
Multiracial	0.92	0.91	0.94
(White)			
Federal poverty level	1.00**	1.00**	1.00**
Overall health		0.83**	0.84**
Autism severity		0.74*	0.74*
Anxiety		0.85	0.86
Depression		1.03	1.05
Behavior problems		0.94	0.94
Adverse childhood experiences			0.91*
Wald	60.54***	27.06***	4.87*

Note: *p < .05, **p < .01, ***p < .001

## Discussion

The results of this study highlight the importance of considering the role of adverse childhood experiences (ACEs) in the lives of children with ASD. The majority (58.7%) of children with ASD in this large national sample had experienced at least one ACE. About one third of the sample had experienced two or more ACEs, while one in ten had experienced four or more. Given the prevalence of sleep problems among children with ASD (Aathira et al., 2017; Reynolds & Malow, 2011; Richdale & Schreck, 2009), we were particularly interested in whether ACEs may be associated with sleep difficulties. Over a third (37%) of children with ASD in the current sample were getting less sleep than recommended by the American Academy of Pediatrics. Our findings revealed that the more ACEs a child with ASD had experienced, the less likely they were to get the recommended number of hours of sleep, even after accounting for other mental health and demographic factors that play a role in sleep duration.

This study is the first to link ACEs with sleep in the autism population. In the general population, adverse events experienced in childhood are associated with adult sleep disorders (Kajeepeta et al., 2015). However, the mechanisms for this association are not fully known. Neuro-physiologically, ACEs likely lead to hyperactivity of the corticotropic-releasing hormone and hypothalamic–pituitary–adrenal (HPA) axis, both of which are critical to regulating sleep (Kajeepeta et al., 2015). On a psychosocial level, Kajeepeta and colleagues (2015) proposed that ACEs disrupt family functioning, which may result in mental health issues or difficulties maintaining a regular household schedule or routine. This, in turn, may contribute to difficulties falling or staying asleep. Our findings suggest that ACEs may help explain the complex and nuanced over-occurrence of sleep problems in this population, as children with ASD are at higher risk for both ACEs and sleep difficulties. Notably, sleep difficulties in the general population are commonly resultant of sleep disordered breathing events (Krakow et al., 2012). It is possible that ACEs may contribute to worsening sleep disordered breathing events in children with ASD. Future research may explore whether autistic children with ACEs have more sleep disordered breathing than children without ACEs.

In our study, race was also significantly associated with sleep duration, as Black and African American children with ASD were 55% less likely to receive the recommended number of hours of sleep as compared to White children. Racial sleep disparities among children have been a consistent finding in previous research, with Black children getting the least amount of sleep (for review, see Guglielmo et al., 2018). Among children with ASD, Elkhatib Smidt and colleagues (2020) found that African American and Black Canadian children were more likely to have sleep problems. Additionally, Black individuals are more likely to have obstructive sleep apnea than White individuals (Dudley & Patel, 2016), which may also explain the findings that Black children were less likely to obtain the recommended amount of sleep. Minoritized families are also less likely to use specialty care such as sleep clinics and thereby less likely to receive treatment (Broder-Fingert et al., 2013). It is important for researchers and clinicians to consider the role of structural and societal factors that may be driving these inequities and acting as barriers to quality sleep in Black children. Our findings highlight the particular importance of supporting Black families in understanding and addressing sleep difficulties in Black autistic children.

The current findings were also consistent with previous research demonstrating that more severe ASD symptoms are associated with poorer sleep (Hollway et al., 2013; Sivertsen et al., 2012; Verhoeff et al., 2018). In fact, recent studies point to a unidirectional association between ASD symptom severity and sleep. Sadikova and colleagues (2022) examined the bi-directional longitudinal relationship between sleep and ASD symptom severity and found that the severity of certain ASD symptoms at baseline can predict future sleep problems. Similarly, Verhoeff and colleagues (2018) also found that ASD symptoms were associated with future sleep symptoms longitudinally. Although our study design was not longitudinal, our theoretical model and findings support the existing link of ASD symptoms severity being a predictor of sleep difficulties. However, the underlying etiology is not clear, especially as sleep disordered breathing can be an undiagnosed confounder in these studies. Other etiologies have been proposed as well, including disruptions of melatonin/cortisol sleep–wake cycles present in both ASD and in sleep difficulties may help explain the link (Tordjman et al., 2015). Others have suggested that the nature of core ASD symptoms such as sensory sensitivity may make it difficult to block out sensory input, thereby making it more difficult to fall and stay asleep (Mazurek & Petroski, 2015). Clinically, it is important to identify the specific ASD-related factors that may interfere with sleep for a particular child in order to develop individualized sleep strategies, especially given the evidence that ASD symptoms can predict sleep difficulties.

Our findings did not show an association between anxiety symptoms and sleep problems among children with ASD, whereas previous literature has shown this association (Mazurek & Petroski, 2015; Rzepecka et al., 2011; Uren et al., 2019). Previous studies have not accounted for adverse childhood experiences. There is likely a complex relationship between anxiety, sleep (including obstructive sleep apnea), and adverse childhood experiences in children with ASD. Future studies should employ multi-method longitudinal methods to further examine these links in autistic individuals.

It should be noted that our findings are preliminary and require further study and replication. Notably, sleep waking in the general population is commonly due to sleep disordered breathing events (Krakow et al., 2012), and sleep disordered breathing is extremely common among individuals who have experienced post-traumatic stress disorder (Krakow et al., 2015). As such, future studies should more fully assess the nature of sleep difficulties, including assessment of sleep disordered breathing, in order to understand the relations among sleep, adverse events, and mental health in this population. A limitation of this study is that all the constructs were assessed only by parent/caregiver report. Issues as complex as sleep, mental health, and ACEs require multi-method and multi-report study. Using direct measures, more comprehensive assessments, and more dimensional approaches to assess sleep, health, mental health, and psychosocial factors would add more nuance, power, and clarity to these preliminary findings. Finally, it is important to note that the analyses conducted were correlational rather than causal. As a result, we are unable to draw any conclusions about causality without further extensive study.

## Conclusion

This study was the first to find a link between adverse childhood experiences (ACEs) and sleep duration (as measured by whether the child gets the recommended number of hours of sleep) in children with ASD. This is an important finding as children with ASD are more vulnerable to both ACEs and sleep difficulties. The underlying reasons for this link are unclear, but may be related to hyperarousal mechanisms in the central nervous system, which have been implicated in both sleep duration and ACEs. Clinically, it is critical to screen all children with ASD for ACEs as they can be at higher risk for experiencing them. It is also recommended that clinicians routinely monitor sleep among all children with ASD, and that sleep difficulties are promptly addressed and treated, particularly among children with a history of ACEs. Finally, African American and Black children are at an especially high risk of experiencing sleep difficulties, and systemic barriers to obtaining adequate sleep should be further explored and addressed.

## Footnotes

1 Given the diversity of preference regarding person first and identity first language, we have chosen to use both when referring to autism spectrum disorder.

## References

[CR1] Aathira, R., Gulati, S., Tripathi, M., Shukla, G., Chakrabarty, B., Sapra, S., Dang, N., Gupta, A., Kabra, M., & Pandey, R. M. (2017). Prevalence of sleep abnormalities in indian children with autism spectrum disorder: A cross-sectional study. *Pediatric Neurology,**74*, 62–67. 10.1016/j.pediatrneurol.2017.05.019 28739359 10.1016/j.pediatrneurol.2017.05.019

[CR2] Anda, R. F., Felitti, V. J., Bremner, J. D., Walker, J. D., Whitfield, Ch., Perry, B. D., Dube, Sh. R., & Giles, W. H. (2006). The enduring effects of abuse and related adverse experiences in childhood. *European Archives of Psychiatry and Clinical Neuroscience,**256*(3), 174–186. 10.1007/s00406-005-0624-4 16311898 10.1007/s00406-005-0624-4 PMC3232061

[CR3] American Psychiatric Association 2013. *Diagnostic and statistical manual of mental disorders: *5th ed. American Psychiatric Association

[CR4] Broder-Fingert, S., Shui, A., Pulcini, C. D., Kurowski, D., & Perrin, J. M. (2013). Racial and ethnic differences in subspecialty service use by children with autism. *Pediatrics,**132*(1), 94–100. 10.1542/peds.2012-3886 23776121 10.1542/peds.2012-3886

[CR5] Charles, L. E., Mnatsakanova, A., Fekedulegn, D., Violanti, J. M., Gu, J. K., & Andrew, M. E. (2022). Associations of adverse childhood experiences (ACEs) with sleep duration and quality: The BCOPS study. *Sleep Medicine,**89*, 166–175. 10.1016/j.sleep.2021.12.011 35026653 10.1016/j.sleep.2021.12.011 PMC8916064

[CR6] Colvonen, P. J., Straus, L. D., Acheson, D., & Gehrman, P. (2019). A review of the relationship between emotional learning and memory, sleep, and PTSD. *Current Psychiatry Reports,**21*(1), 2. 10.1007/s11920-019-0987-2 30661137 10.1007/s11920-019-0987-2 PMC6645393

[CR7] Cronholm, P. F., Forke, C. M., Wade, R., Bair-Merritt, M. H., Davis, M., Harkins-Schwarz, M., Pachter, L. M., & Fein, J. A. (2015). Adverse childhood experiences: Expanding the concept of adversity. *American Journal of Preventive Medicine,**49*(3), 354–361. 10.1016/j.amepre.2015.02.001 26296440 10.1016/j.amepre.2015.02.001

[CR8] Dudley, K. A., & Patel, S. R. (2016). Disparities and genetic risk factors in obstructive sleep apnea. *Sleep Medicine,**18*, 96–102. 10.1016/j.sleep.2015.01.015 26428843 10.1016/j.sleep.2015.01.015 PMC4602395

[CR9] Elkhatib Smidt, S. D., Lu, F., Rao, S. R., Asato, M., & Handen, B. L. (2020). Primary caregiver education level and sleep problems in children with autism spectrum disorder. *Journal of Sleep Research,**29*(5), e12932. 10.1111/jsr.12932 31589359 10.1111/jsr.12932

[CR10] Felitti, V. J., Anda, R. F., Nordenberg, D., Williamson, D. F., Spitz, A. M., Edwards, V., Koss, M. P., & Marks, J. S. (1998). Relationship of childhood abuse and household dysfunction to many of the leading causes of death in adults: The adverse childhood experiences (ACE) study. *American Journal of Preventive Medicine,**14*(4), 245–258. 10.1016/S0749-3797(98)00017-8 9635069 10.1016/s0749-3797(98)00017-8

[CR11] Garbarino, S., Bardwell, W. A., Guglielmi, O., Chiorri, C., Bonanni, E., & Magnavita, N. (2020). Association of anxiety and depression in obstructive sleep apnea patients: A systematic review and meta-analysis. *Behavioral Sleep Medicine,**18*(1), 35–57. 10.1080/15402002.2018.1545649 30453780 10.1080/15402002.2018.1545649

[CR12] Guglielmo, D., Gazmararian, J. A., Chung, J., Rogers, A. E., & Hale, L. (2018). Racial/ethnic sleep disparities in US school-aged children and adolescents: A review of the literature. *Sleep Health,**4*(1), 68–80. 10.1016/j.sleh.2017.09.005 29332684 10.1016/j.sleh.2017.09.005 PMC5771439

[CR13] Han, G. T., Trevisan, D. A., Abel, E. A., Cummings, E. M., Carlos, C., Bagdasarov, A., Kala, S., Parker, T., Canapari, C., & McPartland, J. C. (2022). Associations between sleep problems and domains relevant to daytime functioning and clinical symptomatology in autism: A meta-analysis. *Autism Research,**15*(7), 1249–1260. 10.1002/aur.2758 35635067 10.1002/aur.2758

[CR14] Harder, R., Malow, B. A., Goodpaster, R. L., Iqbal, F., Halbower, A., Goldman, S. E., Fawkes, D. B., Wang, L., Shi, Y., Baudenbacher, F., & Diedrich, A. (2016). Heart rate variability during sleep in children with autism spectrum disorder. *Clinical Autonomic Research,**26*(6), 423–432. 10.1007/s10286-016-0375-5 27491489 10.1007/s10286-016-0375-5 PMC5106315

[CR15] Hollway, J. A., Aman, M. G., & Butter, E. (2013). correlates and risk markers for sleep disturbance in participants of the autism treatment network. *Journal of Autism and Developmental Disorders,**43*(12), 2830–2843. 10.1007/s10803-013-1830-y 23624832 10.1007/s10803-013-1830-y

[CR16] Hoover, D. W., & Kaufman, J. (2018). Adverse childhood experiences in children with autism spectrum disorder. *Current Opinion in Psychiatry,**31*(2), 128–132. 10.1097/YCO.0000000000000390 29206686 10.1097/YCO.0000000000000390 PMC6082373

[CR17] Hundley, R. J., Shui, A., & Malow, B. A. (2016). Relationship between subtypes of restricted and repetitive behaviors and sleep disturbance in autism spectrum disorder. *Journal of Autism and Developmental Disorders,**46*(11), 3448–3457. 10.1007/s10803-016-2884-4 27511195 10.1007/s10803-016-2884-4

[CR18] Kajeepeta, S., Gelaye, B., Jackson, C. L., & Williams, M. A. (2015). Adverse childhood experiences are associated with adult sleep disorders: A systematic review. *Sleep Medicine,**16*(3), 320–330. 10.1016/j.sleep.2014.12.013 25777485 10.1016/j.sleep.2014.12.013 PMC4635027

[CR19] Krakow, B. (2022). The dirty (not-so-little) secret about “clean” insomnia. *Journal of Clinical Sleep Medicine,**18*(12), 2877–2878. 10.5664/jcsm.10276 36453604 10.5664/jcsm.10276 PMC9713926

[CR20] Krakow, U., & V. A., Moore, B. A., & McIver, N. D. (2015). Posttraumatic stress disorder and sleep-disordered breathing: A review of comorbidity research. *Sleep Medicine Reviews,**24*, 37–45. 10.1016/j.smrv.2014.11.001 25644985 10.1016/j.smrv.2014.11.001

[CR21] Krakow, B., Romero, E., Ulibarri, V. A., & Kikta, S. (2012). Prospective assessment of nocturnal awakenings in a case series of treatment-seeking chronic insomnia patients: A pilot study of subjective and objective causes. *Sleep,**35*(12), 1685–1692. 10.5665/sleep.2244 23204611 10.5665/sleep.2244 PMC3490361

[CR22] Krakow, B., McIver, N. D., Ulibarri, V. A., Krakow, J., & Schrader, R. M. (2019). Prospective randomized controlled trial on the efficacy of continuous positive airway pressure and adaptive servo-ventilation in the treatment of chronic complex insomnia. *EClinicalMedicine,**13*, 57–73. 10.1016/j.eclinm.2019.06.011 31517263 10.1016/j.eclinm.2019.06.011 PMC6734001

[CR23] Maenner, M. J. (2021). Prevalence and characteristics of autism spectrum disorder among children aged 8 years–autism and developmental disabilities monitoring network, 11 sites united states, 2018. *MMWR. Surveillance Summaries,**70*, 1–16. 10.15585/mmwr.ss7011a1 10.15585/mmwr.ss7011a1 PMC8639024 34855725

[CR24] Mazurek, M. O., & Petroski, G. F. (2015). Sleep problems in children with autism spectrum disorder: Examining the contributions of sensory over-responsivity and anxiety. *Sleep Medicine,**16*(2), 270–279. 10.1016/j.sleep.2014.11.006 25600781 10.1016/j.sleep.2014.11.006

[CR25] Mazurek, M. O., & Sohl, K. (2016). Sleep and behavioral problems in children with autism spectrum disorder. *Journal of Autism and Developmental Disorders,**46*(6), 1906–1915. 10.1007/s10803-016-2723-7 26823076 10.1007/s10803-016-2723-7

[CR26] Monnat, S. M., & Chandler, R. F. (2015). Long-term physical health consequences of adverse childhood experiences. *The Sociological Quarterly,**56*(4), 723–752. 10.1111/tsq.12107 26500379 10.1111/tsq.12107 PMC4617302

[CR27] Murata, E., Mohri, I., Kato-Nishimura, K., Iimura, J., Ogawa, M., Tachibana, M., Ohno, Y., & Taniike, M. (2017). Evaluation of behavioral change after adenotonsillectomy for obstructive sleep apnea in children with autism spectrum disorder. *Research in Developmental Disabilities,**65*, 127–139. 10.1016/j.ridd.2017.04.012 28514706 10.1016/j.ridd.2017.04.012

[CR28] Reynolds, A. M., & Malow, B. A. (2011). Sleep and autism spectrum disorders. *Pediatric Clinics of North America,**58*(3), 685–698. 10.1016/j.pcl.2011.03.009 21600349 10.1016/j.pcl.2011.03.009

[CR29] Richdale, A. L., & Schreck, K. A. (2009). Sleep problems in autism spectrum disorders: Prevalence, nature, & possible biopsychosocial aetiologies. *Sleep Medicine Reviews,**13*(6), 403–411. 10.1016/j.smrv.2009.02.003 19398354 10.1016/j.smrv.2009.02.003

[CR30] Rigles, B. (2021). Trajectories of adverse childhood experiences among children with autism. *Research in Autism Spectrum Disorders,**89*, 101876. 10.1016/j.rasd.2021.101876

[CR31] Rzepecka, H., McKenzie, K., McClure, I., & Murphy, S. (2011). Sleep, anxiety and challenging behaviour in children with intellectual disability and/or autism spectrum disorder. *Research in Developmental Disabilities,**32*(6), 2758–2766. 10.1016/j.ridd.2011.05.034 21700417 10.1016/j.ridd.2011.05.034

[CR32] Sadikova, E., Dovgan, K., & Mazurek, M. O. (2022). Longitudinal examination of sleep problems and symptom severity in children with autism spectrum disorder. *Journal of Autism and Developmental Disorders*. 10.1007/s10803-021-05401-1 35384626 10.1007/s10803-021-05401-1

[CR33] Santapuram, P., Chen, H., Weitlauf, A. S., Ghani, M. O. A., & Whigham, A. S. (2022). Investigating differences in symptomatology and age at diagnosis of obstructive sleep apnea in children with and without autism. *International Journal of Pediatric Otorhinolaryngology,**158*, 111191. 10.1016/j.ijporl.2022.111191 35636082 10.1016/j.ijporl.2022.111191

[CR34] Schreck, K. A., & Richdale, A. L. (2020). Sleep problems, behavior, and psychopathology in autism: Inter-relationships across the lifespan. *Current Opinion in Psychology,**34*, 105–111. 10.1016/j.copsyc.2019.12.003 31918238 10.1016/j.copsyc.2019.12.003

[CR35] Sivertsen, B., Posserud, M.-B., Gillberg, C., Lundervold, A. J., & Hysing, M. (2012). Sleep problems in children with autism spectrum problems: A longitudinal population-based study. *Autism,**16*(2), 139–150. 10.1177/1362361311404255 21478225 10.1177/1362361311404255

[CR36] Soke, G. N., Maenner, M. J., Christensen, D., Kurzius-Spencer, M., & Schieve, L. A. (2018). Prevalence of co-occurring medical and behavioral conditions/symptoms among 4- and 8-year-old children with autism spectrum disorder in selected areas of the united states in 2010. *Journal of Autism and Developmental Disorders,**48*(8), 2663–2676. 10.1007/s10803-018-3521-1 29524016 10.1007/s10803-018-3521-1 PMC6041136

[CR37] Taylor, J. L., & Corbett, B. A. (2014). A review of rhythm and responsiveness of cortisol in individuals with autism spectrum disorders. *Psychoneuroendocrinology,**49*, 207–228. 10.1016/j.psyneuen.2014.07.015 25108163 10.1016/j.psyneuen.2014.07.015 PMC4165710

[CR38] Tomkies, A., Johnson, R. F., Shah, G., Caraballo, M., Evans, P., & Mitchell, R. B. (2019). Obstructive sleep apnea in children with autism. *Journal of Clinical Sleep Medicine,**15*(10), 1469–1476. 10.5664/jcsm.7978 31596212 10.5664/jcsm.7978 PMC6778353

[CR39] Tordjman, S., Davlantis, K. S., Georgieff, N., Geoffray, M.-M., Speranza, M., Anderson, G. M., Xavier, J., Botbol, M., Oriol, C., Bellissant, E., Vernay-Leconte, J., Fougerou, C., Hespel, A., Tavenard, A., Cohen, D., Kermarrec, S., Coulon, N., Bonnot, O., & Dawson, G. (2015). Autism as a disorder of biological and behavioral rhythms: Toward new therapeutic perspectives. *Frontiers in Pediatrics,**3*, 1. 10.3389/fped.2015.00001 25756039 10.3389/fped.2015.00001 PMC4337381

[CR40] United States Census Bureau (2016) *2016 NSCH Data Release* Census.Gov. https://www.census.gov/data/datasets/2016/demo/nsch/nsch2016.html

[CR41] United States Census Bureau (2017) *2017 NSCH Data Release*. Census.Gov. https://www.census.gov/data/datasets/2017/demo/nsch/nsch2017.html

[CR42] United States Census Bureau (2018) *2018 NSCH Data Release*. Census.Gov. https://www.census.gov/data/datasets/2018/demo/nsch/nsch2018.html

[CR43] United States Census Bureau (2019) *2019 NSCH Data Release*. Census.Gov. https://www.census.gov/data/datasets/2019/demo/nsch/nsch2019.html

[CR44] Uren, J., Richdale, A. L., Cotton, S. M., & Whitehouse, A. J. O. (2019). Sleep problems and anxiety from 2 to 8 years and the influence of autistic traits: A longitudinal study. *European Child & Adolescent Psychiatry,**28*(8), 1117–1127. 10.1007/s00787-019-01275-y 30659385 10.1007/s00787-019-01275-y

[CR45] Vadukapuram, R., Shah, K., Ashraf, S., Srinivas, S., Elshokiry, A. B., Trivedi, C., Mansuri, Z., & Jain, S. (2022). Adverse childhood experiences and their impact on sleep in adults: A systematic review. *The Journal of Nervous and Mental Disease,**210*(6), 397–410. 10.1097/NMD.0000000000001480 35640064 10.1097/NMD.0000000000001480

[CR46] van der Kolk, B. A. (2003). The neurobiology of childhood trauma and abuse. *Child and Adolescent Psychiatric Clinics,**12*(2), 293–317. 10.1016/S1056-4993(03)00003-8 10.1016/s1056-4993(03)00003-8 12725013

[CR47] Vargas, I., Nguyen, A. M., Muench, A., Bastien, C. H., Ellis, J. G., & Perlis, M. L. (2020). Acute and chronic insomnia: What has time and/or hyperarousal got to do with it? *Brain Sciences,**10*(2), 71. 10.3390/brainsci10020071 32013124 10.3390/brainsci10020071 PMC7071368

[CR48] Verhoeff, M. E., Blanken, L. M. E., Kocevska, D., Mileva-Seitz, V. R., Jaddoe, V. W. V., White, T., Verhulst, F., Luijk, M. P. C. M., & Tiemeier, H. (2018). The bidirectional association between sleep problems and autism spectrum disorder: A population-based cohort study. *Mol Autism.,**9*(1), 8. 10.1186/s13229-018-0194-8 29423134 10.1186/s13229-018-0194-8 PMC5791216

[CR49] Zeidan, J., Fombonne, E., Scorah, J., Ibrahim, A., Durkin, M. S., Saxena, S., Yusuf, A., Shih, A., & Elsabbagh, M. (2022). Global prevalence of autism: A systematic review update. *Autism Research,**1*(13), 778–790. 10.1002/aur.2696 10.1002/aur.2696 PMC9310578 35238171

